# DEVELOPMENT OF EPIGENETIC MEASURES FOR GEROSCIENCE CLINICAL TRIALS

**DOI:** 10.1093/geroni/igz038.2732

**Published:** 2019-11-08

**Authors:** Morgan E Levine

**Affiliations:** Yale University School of Medicine, New Haven, Connecticut, United States

## Abstract

One of the major goals of the NIA is to oversee development of biomarkers of aging. In recent years, DNA methylation has emerged as a promising avenue from which to quantify biological age. We and others have shown that these measures track age across various tissues and cells, and further deviations between chronological and “epigenetic age” have been shown to confer risk for various aging outcomes. However, the usefulness of these measures will depend on both their modifiability and ability to capture known targetable hallmarks of aging. Using DNA methylation data from cell line experiments, we have recently generated epigenetic predictors of cellular senescence for both human and mouse that when assessed in vivo from bulk samples, show age-related increases and are associated with aging outcomes. In moving forward, measures such as these may serve as promising surrogate endpoints for assessing efficacy of senolytic drugs and/or other anti-aging therapeutics.

